# Body composition and blood pressure: Haemodynamic links in young men and women

**DOI:** 10.1113/EP093982

**Published:** 2026-11-11

**Authors:** Domonkos Cseh, Jessica E. Middlemiss, Shikai Yu, Gisele J. Bentley, Abbie R. Williams, Yasmin, Barry J. McDonnell, Christopher J. A. Pugh, Ian B. Wilkinson, Carmel M. McEniery

**Affiliations:** ^1^ Division of Experimental Medicine and Immunotherapeutics University of Cambridge Cambridge UK; ^2^ Department of Physiology Semmelweis University Budapest Hungary; ^3^ Department of Cardiology School of Medicine Shanghai Tenth People's Hospital, Tongji University Shanghai China; ^4^ Centre for Cardiovascular Research, Innovation and Development Cardiff Metropolitan University Cardiff UK

**Keywords:** blood pressure, body composition, cardiac output, fat‐free mass, fat mass, haemodynamics, peripheral vascular resistance, pulse wave velocity

## Abstract

The aim of this study was to explore the sex‐specific haemodynamic links between body composition parameters and systolic and diastolic blood pressure (SBP and DBP, respectively) in adults free of cardiovascular disease and not receiving cardiovascular medications. Body mass index (BMI), fat mass (FM), fat‐free mass (FFM), SBP and DBP, cardiac output (CO), peripheral vascular resistance (PVR) and aortic pulse wave velocity (PWV) were determined in 235 men and 192 women aged 18–40 years. Multivariable linear regression and single‐ as well as multiple‐mediator models were used to assess haemodynamic mechanisms underlying the associations between body composition and blood pressure. BMI showed positive associations with SBP and DBP in both sexes (*P *< 0.001 for all). In men, FFM was the main body composition correlate of SBP (*P *< 0.001), with the positive association driven in part by CO and partially offset by opposing effects through PVR. FM was the dominant body composition correlate of DBP in men and both SBP and DBP in women (*P *< 0.001 for all), with the positive associations mediated by PVR and PWV in both sexes, and additionally by heart rate in the FM–DBP association in men. Changes in FFM may affect SBP predominantly through CO in young men. Changes in FM may affect DBP in young men and both SBP and DBP in young women, predominantly through vascular mechanisms. These findings provide important insights into sex differences in the physiology of adiposity‐related BP changes in young adults and may help inform sex‐specific strategies for the management of adiposity‐related hypertension.

## Abstract (highlights)

- **What is the central question of this study?**
  What are the sex‐specific haemodynamic pathways linking body composition parameters to systolic and diastolic blood pressure in young adults free from cardiovascular disease and cardiovascular medication?
- **What is the main finding and its importance?**
  Fat‐free mass is positively associated with systolic blood pressure in men, predominantly mediated by cardiac output, whereas fat mass is positively associated with diastolic blood pressure in men and with both systolic and diastolic blood pressure in women, predominantly mediated by vascular mechanisms. These findings may help refine sex‐specific strategies for the management of hypertension related to adiposity.

## 1 INTRODUCTION

Hypertension is one of the most important public health challenges worldwide, and represents the leading modifiable risk factor for death and disability globally (Brauer et al., 2024). Obesity is another major risk factor for non‐communicable diseases, and its increasing prevalence has reached pandemic levels (Bluher, 2019). Hypertension and obesity share a well‐known association, but despite extensive evidence linking the two conditions (Garrison et al., 1987; Zhang et al., 2022), the haemodynamic mechanisms underlying this association remain poorly understood. Several biological mechanisms have been implicated in obesity‐related hypertension, including activation of the renin–angiotensin–aldosterone and sympathetic nervous systems, inflammation, oxidative stress and endothelial dysfunction (Clayton et al., 2023; DeMarco et al., 2014; Hall et al., 2015; Parvanova et al., 2024). These mechanisms may ultimately influence blood pressure (BP) through cardiac and vascular haemodynamic alterations.

Previous epidemiological studies have shown that there is a positive association between body mass index (BMI) and BP, and that this relationship is steeper in younger individuals (Bann et al., 2021; Linderman et al., 2018). This suggests that understanding the mechanisms underlying the association between body size and BP may be particularly important at a young age. Our group previously demonstrated that the haemodynamic mechanisms underlying increased systolic BP differ according to adiposity, estimated using BMI, and sex in young adults (Middlemiss et al., 2016). In that study, we focused on the major haemodynamic determinants of arterial BP such as cardiac output (CO), stroke volume (SV), peripheral vascular resistance (PVR) and aortic pulse wave velocity (PWV). However, BMI is an imperfect surrogate of adiposity, as it does not fully reflect body composition (Oikonomou & Antoniades, 2019). Distinguishing between fat mass (FM) and fat‐free mass (FFM) may be particularly important when examining haemodynamic mechanisms, as these tissue compartments have different metabolic and circulatory requirements (Cunningham, 1991; Wang et al., 2010).

Collis et al. examined the relationship between body composition and cardiac haemodynamic parameters, such as SV and CO, in participants of the Strong Heart Study (Collis et al., 2001). They showed that SV and CO were more strongly related to FFM than adipose mass in both sexes. However, the age range of their population was wide (45–74 years), their analyses were not stratified by age, and participants taking cardiovascular medications were not excluded. Furthermore, young individuals – among whom sex‐related differences in haemodynamic mechanisms underlying elevated BP may be particularly pronounced (Cseh et al., 2025; Middlemiss et al., 2016; Nardin et al., 2018) – were not included. Other studies explored the links between body composition and blood pressure (Zhao et al., 2022), and demonstrated that an increase in body fat is associated with a higher risk of developing sustained hypertension (Markus et al., 2010), while findings regarding the relationship between lean body mass indices and BP remain controversial (Li et al., 2018; Ye et al., 2018).

Notably, none of the mentioned studies focused on the haemodynamic mechanisms linking body composition to BP in young adults. Therefore, the aim of the present study was to investigate these mechanisms separately in young men and women, in a population free of cardiovascular disease and cardiovascular medication. We hypothesized that the haemodynamic mechanisms underlying the associations between body composition and BP would differ between young men and women. Understanding the mechanisms behind the development of obesity‐related hypertension during this critical time period may provide an important opportunity for disease prevention and early intervention to achieve better long‐term cardiovascular outcomes (Scott et al., 2025).

## 2 METHODS

### 2.1 Ethical approval

The study was a cross‐sectional analysis of data collected as part of the study titled ‘Influence of Age, Weight and Ethnic background on blood pressure’ (AWE study). It was conducted in accordance with the *Declaration of Helsinki* (2024) and received approval from the West Midlands – Coventry and Warwickshire Research Ethics Committee (REC reference number: 16/WM/0485). Written informed consent was received from each participant prior to any study‐related investigations.

### 2.2 Participants

Participants aged 18–40 years (235 men and 192 women) with available body composition and supine BP data and without a history of cardiovascular disease or current use of cardiovascular medication were included in the present analysis.

### 2.3 Protocol

Measurements were performed in a temperature‐controlled (22–24°C), quiet room. First, height, weight and body composition were assessed. Following at least 5 min of seated rest, brachial BP was measured. After a further 10 min of supine rest, brachial BP was measured again and pressure waveforms were recorded from the radial, carotid and femoral arteries. SV and CO were subsequently measured, and PVR was then calculated.

### 2.4 Body composition measurement

FM, FFM, fat mass percentage (FM%), fat‐free mass percentage (FFM%) and basal metabolic rate (BMR) were assessed using bioelectrical impedance scales (Tanita BC‐418, Tanita Corporation, Tokyo, Japan).

### 2.5 Haemodynamic measurements

Seated and supine brachial systolic and diastolic BP (SBP and DBP, respectively) were measured using a validated oscillometric device (HEM‐907; Omron Corporation, Kyoto, Japan). A minimum of three consecutive BP measurements were performed, and the average of the final two measurements was used in the analysis. A high‐fidelity micromanometer (SPC‐301; Millar Instruments, Pearland, TX, USA) was used to record radial artery pressure waveforms, and using the SphygmoCor system (AtCor Medical, Sydney, Australia), mean arterial pressure (MAP) was obtained by integration of the pressure waveform. Then, sequentially recorded, ECG‐gated carotid and femoral artery waveforms were used to measure carotid–femoral (aortic) PWV, as described previously (Wilkinson et al., 1998). SV and CO were measured using a non‐invasive, validated, inert gas rebreathing technique (Gabrielsen et al., 2002; Middlemiss et al., 2019). Briefly, subjects breathed from a gas mixture (1% SF_6_, 5% N_2_O and 94% O_2_) with a fixed breathing rate for 20 s, and an infrared photoacoustic gas analyser embedded in the device (Innocor; Innovision A/S, Glamsbjerg, Denmark) continuously sampled and analysed the expired gases at the mouthpiece. PVR was then calculated using the following formula: PVR (dyn s cm^−5^) = MAP (mmHg) × 80/CO (L/min). The factor of 80 is the standard conversion factor from Wood units (mmHg min L^−1^) to dyn s cm^−5^ (1 mmHg = 1333.22 dyn cm^−2^; 1 L/min = 16.67 cm^3^ s^−1^).

### 2.6 Statistical analysis

Data analysis was performed using SPSS version 29 (IBM Corp., Armonk, NY, USA). Data are presented as means ± SD for normally distributed variables and median (interquartile range) for variables with skewed distributions. *P *< 0.05 was considered significant for all analyses.

Comparisons between men and women were performed using Student's independent‐samples *t*‐test for normally distributed variables, and the Mann‐Whitney *U*‐test for non‐normally distributed variables. All subsequent analyses were performed separately for men and women.

The associations between BMI and supine SBP and DBP were examined using simple linear regression. Age‐adjusted multivariable linear regression models were used to examine the relationship between body composition parameters (FM and FFM) and supine BP parameters (SBP and DBP), as well as between body composition parameters and other haemodynamic variables [supine CO, SV, heart rate (HR), PVR and PWV]. Age, FM and FFM were included simultaneously in these models. Unique *R* ^2^ values were derived from squared semi‐partial correlations.

Based on the results of these multivariable analyses, single‐mediator models were used to examine the indirect effects of relevant haemodynamic pathways between selected body composition parameter and selected BP parameter (Figure 1a). A body composition parameter was included as the independent variable in mediation analysis if it was significantly associated with the selected BP parameter in the multivariable linear regression models. Haemodynamic variables were selected as candidate mediators based on physiological relevance and significant associations with the body composition parameter of interest in the multivariable linear regression models.

**FIGURE 1:**
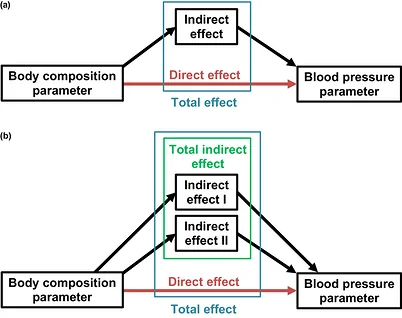
Single‐mediator models (a) and multiple‐mediator models (b) in our analyses.

Based on the results of the single‐mediator models, parallel multiple‐mediator models were constructed to assess the combined and individual indirect effects of selected haemodynamic pathways between selected body composition parameters and selected BP parameters (Figure 1b). Mediators were included in the parallel models if they demonstrated a significant indirect effect in the corresponding single‐mediator models. Mediation analyses were conducted using the PROCESS macro (version 5.0; Model 4) by Andrew F. Hayes (Hayes, 2022). Point estimates and 95% confidence intervals for total and direct effects were obtained using ordinary least squares regression, with 95% confidence intervals for indirect effects derived from non‐parametric bootstrap resampling (5000 samples).

No formal adjustment for multiple testing was applied. Our analyses were performed according to a physiologically guided analytical framework rather than representing unrestricted exploratory testing.

As a sensitivity analysis, all multivariable and mediation analyses were repeated with additional adjustment for height to distinguish the effects of body composition from those of stature.

## 3 RESULTS

Table 1 shows the anthropometric, body composition and haemodynamic characteristics of the study participants, stratified by sex. Age and BMI did not differ significantly between men and women, although both comparisons approached conventional levels of significance. Height and weight were significantly higher in men than women. While FM and FM% were significantly higher in women, FFM, FFM% and BMR were significantly higher in men. Both seated and supine SBP, as well as supine MAP, were significantly higher in men, whereas supine HR was significantly lower compared with women. No significant differences were observed in seated and supine DBP values between the two groups. While men exhibited higher CO, SV and PWV, PVR was higher in women.

**TABLE 1:** Anthropometric, body composition and haemodynamic parameters of the participants.

	Men (*n* = 235)	Women (*n* = 192)	*P*
**Age (years)**	28 (24–34)	27 (23–31)	0.052
**Height (cm)**	179 ± 8	166 ± 7	<0.001
**Weight (kg)**	78.0 (71.7–89.7)	65.0 (56.4–78.8)	<0.001
**Body mass index (kg/m^2^)**	24.8 (22.5–27.6)	23.5 (20.7–28.2)	0.051
**Fat mass (kg)**	13.7 (9.5–19.7)	19.0 (13.7–29.0)	<0.001
**Fat free mass (kg)**	65.5 ± 9.3	46.1 ± 5.5	<0.001
**Fat mass percentage (%)**	18.5 ± 7.4	31.0 ± 9.0	<0.001
**Fat free mass percentage (%)**	81.5 ± 7.4	69.0 ± 9.0	<0.001
**Basal metabolic rate (kJ/day)**	8070 ± 1197	5964 ± 741	<0.001
**Seated systolic blood pressure (mmHg)**	129 ± 13	116 ± 15	<0.001
**Seated diastolic blood pressure (mmHg)**	78 ± 10	76 ± 12	0.204
**Supine systolic blood pressure (mmHg)**	125 ± 12	115 ± 15	<0.001
**Supine diastolic blood pressure (mmHg)**	72 ± 11	72 ± 12	0.953
**Supine mean arterial pressure (mmHg)**	88 ± 12	85 ± 14	0.035
**Supine heart rate (bpm)**	61 ± 10	65 ± 11	<0.001
**Supine cardiac output (L/min)**	7.3 ± 1.7	6.1 ± 1.4	<0.001
**Supine stroke volume (mL)**	112 ± 28	88 ± 21	<0.001
**Supine peripheral vascular resistance (dyn s cm^−5^)**	1044 ± 337	1188 ± 321	<0.001
**Supine pulse wave velocity (m/s)**	5.8 (5.2–6.7)	5.2 (4.6–5.8)	<0.001

Data are means ± SD or medians (interquartile range).

BMI was positively associated with SBP in both men and women, with a slightly higher unstandardized regression coefficient in men (B = 1.06 mmHg per kg/m^2^ in men and B = 0.97 mmHg per kg/m^2^ in women; *P *< 0.001 for both sexes). Similar associations were observed between BMI and DBP (B = 1.15 mmHg per kg/m^2^ in men and B = 0.94 mmHg per kg/m^2^ in women; *P *< 0.001 for both sexes).

The multivariable linear regression models examining the haemodynamic correlates of body composition parameters are presented in Tables 2 and 3 for men and women, respectively. In men, FFM showed a significant positive association with SBP independent of FM, whereas FM was not independently associated with SBP. In contrast, FM showed a significant positive association with DBP, independent of FFM, and FFM was not independently associated with DBP. FFM was strongly and positively associated with CO and SV, and inversely associated with HR and PVR. FM showed significant positive associations with HR, PVR and PWV.

**TABLE 2:** Multivariable linear regression models exploring the links between body composition parameters and systolic blood pressure (A), diastolic blood pressure (B), cardiac output (C), stroke volume (D), heart rate (E), peripheral vascular resistance (F), and pulse wave velocity (G) in men.

	B	β	Unique *R* ^2^ (%)	*P*
**A. Systolic blood pressure**				
Adjusted *R* ^2 ^= 0.155				
Age	0.326	0.161	2.0	0.018
Fat mass	0.153	0.107	0.8	0.142
***Fat‐free mass***	***0.359***	***0.270***	***6.1***	***<0.001***
**B. Diastolic blood pressure**				
Adjusted *R* ^2 ^= 0.352				
***Age***	***0.657***	***0.381***	***11.5***	***<0.001***
***Fat mass***	***0.417***	***0.343***	***8.0***	***<0.001***
Fat‐free mass	−0.075	−0.066	0.4	0.253
**C. Cardiac output**				
Adjusted *R* ^2 ^= 0.274				
***Age***	−***0.089***	−***0.325***	***8.3***	***<0.001***
Fat mass	−0.005	−0.027	0.0	0.695
***Fat‐free mass***	***0.088***	***0.485***	***19.5***	***<0.001***
**D. Stroke volume**				
Adjusted *R* ^2 ^= 0.268				
***Age***	−***0.985***	−***0.214***	***3.6***	***<0.001***
Fat mass	−0.392	−0.121	1.0	0.078
***Fat‐free mass***	***1.627***	***0.536***	***23.8***	***<0.001***
**E. Heart rate**				
Adjusted *R* ^2 ^= 0.026				
Age	−0.120	−0.075	0.4	0.307
***Fat mass***	***0.251***	***0.222***	***3.4***	***0.005***
***Fat‐free mass***	−***0.152***	−***0.144***	***1.7***	***0.044***
**F. Peripheral vascular resistance**				
Adjusted *R* ^2 ^= 0.317				
***Age***	***20.417***	***0.431***	***14.7***	***<0.001***
***Fat mass***	***5.169***	***0.155***	***1.6***	***0.020***
***Fat‐free mass***	−***12.906***	−***0.413***	***14.1***	***<0.001***
**G. Pulse wave velocity**				
Adjusted *R* ^2 ^= 0.292				
***Age***	***0.070***	***0.413***	***13.8***	***<0.001***
***Fat mass***	***0.023***	***0.196***	***2.7***	***0.004***
Fat‐free mass	0.007	0.060	0.3	0.326

Significant predictors are shown in bold italics. B, unstandardised regression coefficient; β, standardised regression coefficient.

**TABLE 3:** Multivariable linear regression models exploring the links between body composition parameters and systolic blood pressure (A), diastolic blood pressure (B), cardiac output (C), stroke volume (D), heart rate (E), peripheral vascular resistance (F), and pulse wave velocity (G) in women.

	B	β	Unique *R* ^2^ (%)	*P*
**A. Systolic blood pressure**				
Adjusted *R* ^2 ^= 0.168				
Age	0.214	0.083	0.6	0.226
***Fat mass***	***0.440***	***0.366***	***6.3***	***<0.001***
Fat‐free mass	0.126	0.047	0.1	0.619
**B. Diastolic blood pressure**				
Adjusted *R* ^2 ^= 0.238				
***Age***	***0.275***	***0.129***	***1.6***	***0.049***
***Fat mass***	***0.531***	***0.535***	***13.4***	***<0.001***
Fat‐free mass	−0.280	−0.126	0.8	0.163
**C. Cardiac output**				
Adjusted *R* ^2 ^= 0.196				
Age	−0.026	−0.110	1.1	0.104
Fat mass	−0.013	−0.112	0.6	0.232
***Fat‐free mass***	***0.131***	***0.519***	***13.4***	***<0.001***
**D. Stroke volume**				
Adjusted *R* ^2 ^= 0.180				
Age	0.053	0.014	0.0	0.835
***Fat mass***	−***0.639***	−***0.362***	***6.3***	***<0.001***
***Fat‐free mass***	***2.422***	***0.612***	***18.7***	***<0.001***
**E. Heart rate**				
Adjusted *R* ^2 ^= 0.085				
Age	−0.172	−0.092	0.8	0.198
***Fat mass***	***0.392***	***0.453***	***9.6***	***<0.001***
***Fat‐free mass***	−***0.505***	***0.191***	***3.3***	***0.009***
**F. Peripheral vascular resistance**				
Adjusted *R* ^2 ^= 0.145				
Age	8.056	0.143	1.9	0.043
***Fat mass***	***10.507***	***0.394***	***7.5***	***<0.001***
***Fat‐free mass***	−***29.198***	−***0.490***	***12.1***	***<0.001***
**G. Pulse wave velocity**				
Adjusted *R* ^2 ^= 0.154				
***Age***	***0.032***	***0.213***	***4.3***	***0.003***
***Fat mass***	***0.022***	***0.294***	***4.2***	***0.003***
Fat‐free mass	0.004	0.025	0.0	0.798

Significant predictors are shown in bold italics.B, unstandardised regression coefficient; β, standardised regression coefficient.

In women, FM was the primary independent positive correlate of both SBP and DBP. FM showed no independent association with CO, a negative association with SV, and positive associations with HR, PVR and PWV. FFM was positively associated with CO and SV, negatively associated with HR and PVR, and showed no independent association with PWV.

The results of the mediation analyses showing the association between the dominant body composition parameter and SBP are presented in Tables 4 and 5 for men and women, respectively. In men, single‐mediator models showed that CO positively, and PVR negatively, mediated the FFM–SBP association, whereas indirect effects via SV and HR were non‐significant. In the multiple‐mediator model, the opposing indirect effects via cardiac output and PVR were confirmed, but the total indirect effect was non‐significant, with a significant direct effect remaining. In women, HR, PVR and PWV positively mediated the FM–SBP association in single‐mediator models. There was no significant mediation via SV. In the multiple‐mediator model, PVR and PWV remained significant mediators, and the direct effect was not significant, consistent with full mediation of the association between FM and SBP.

**TABLE 4:** Mediation analysis of the association between fat‐free mass and systolic blood pressure via cardiac output (CO; A), stroke volume (SV; B), heart rate (HR; C), peripheral vascular resistance (PVR; D), and via cardiac output and peripheral vascular resistance (E) in men.

	Effect (mmHg/kg)	95% CI	*P*
**A. Mediator: cardiac output**			
***Total effect***	***0.362***	***0.188–0.536***	***<0.001***
***Direct effect***	***0.245***	***0.051–0.439***	***0.014***
***Indirect effect (CO)***	***0.117***	***0.021–0.218***	***—***
**B. Mediator: stroke volume**			
***Total effect***	***0.362***	***0.188–0.536***	***<0.001***
***Direct effect***	***0.375***	***0.174–0.576***	***<0.001***
Indirect effect (SV)	−0.014	−0.114–0.077	***—***
**C. Mediator: heart rate**			
***Total effect***	***0.362***	***0.189–0.536***	***<0.001***
***Direct effect***	***0.390***	***0.217–0.563***	***<0.001***
Indirect effect (HR)	−0.028	−0.073–0.001	***—***
**D. Mediator: peripheral vascular resistance**			
***Total effect***	***0.360***	***0.186–0.534***	***<0.001***
***Direct effect***	***0.517***	***0.332–0.702***	***<0.001***
***Indirect effect (PVR)***	−***0.157***	−***0.256–***−***0.063***	***—***
**E. Mediators: cardiac output and peripheral vascular resistance**			
***Total effect***	***0.360***	***0.186–0.534***	***<0.001***
***Direct effect***	***0.308***	***0.158–0.459***	***<0.001***
Total indirect effect	0.052	−0.090–0.189	***—***
***Indirect effect (CO)***	***0.681***	***0.499–0.880***	***—***
***Indirect effect (PVR)***	−***0.629***	−***0.820–***−***0.466***	***—***

All models were adjusted for age and fat mass. Significant effects are shown in bold italics. The indirect effects are considered significant when the corresponding confidence intervals do not include zero. CI, confidence interval.

**TABLE 5:** Mediation analysis of the association between fat mass and systolic blood pressure via stroke volume (SV; A), heart rate (HR; B), peripheral vascular resistance (PVR; C), pulse wave velocity (PWV; D), and via heart rate, peripheral vascular resistance and pulse wave velocity (E) in women.

	Effect (mmHg/kg)	95% CI	*P*
**A. Mediator: stroke volume**			
***Total effect***	***0.439***	***0.209–0.669***	***<0.001***
***Direct effect***	***0.434***	***0.195–0.674***	***<0.001***
Indirect effect (SV)	0.001	−0.049–0.081	—
**B. Mediator: heart rate**			
***Total effect***	***0.435***	***0.205–0.664***	***<0.001***
***Direct effect***	***0.361***	***0.121–0.600***	***0.003***
***Indirect effect (HR)***	***0.074***	***0.002–0.168***	—
**C. Mediator: peripheral vascular resistance**			
***Total effect***	***0.423***	***0.193–0.653***	***<0.001***
***Direct effect***	***0.222***	***0.004–0.440***	***0.047***
***Indirect effect (PVR)***	***0.200***	***0.083–0.371***	—
**D. Mediator: pulse wave velocity**			
***Total effect***	***0.356***	***0.113–0.559***	***0.004***
Direct effect	0.152	−0.056–0.360	0.152
***Indirect effect (PWV)***	***0.205***	***0.075–0.371***	—
**E. Mediators: heart rate, peripheral vascular resistance and pulse wave velocity**			
***Total effect***	***0.354***	***0.108–0.599***	***0.005***
Direct effect	0.012	−0.201–0.224	0.914
***Total indirect effect***	***0.342***	***0.172–0.554***	—
Indirect effect (HR)	0.018	−0.054–0.098	—
***Indirect effect (PVR)***	***0.162***	***0.060–0.310***	—
***Indirect effect (PWV)***	***0.163***	***0.052–0.310***	—

All models were adjusted for age and fat‐free mass. Significant effects are shown in bold italics. The indirect effects are considered significant when the corresponding confidence intervals do not include zero. CI, confidence interval.

Tables 6 and 7 present the results of the mediation analyses showing the association between the dominant body composition parameter and DBP for men and women, respectively. In men, single‐mediator models showed that HR, PVR and PWV positively mediated the FM–DBP association. In the multiple‐mediator model, all three variables showed a positive indirect effect, with PVR having the largest effect. A significant direct effect remained in this model. In women, HR, PVR and PWV positively mediated the FM–DBP association in single‐mediator models. There was no significant mediation via SV. The multiple‐mediator model showed that PVR and PWV were positive significant independent mediators, and the direct effect was also significant.

**TABLE 6:** Mediation analysis of the association between fat mass and diastolic blood pressure via heart rate (HR; A), peripheral vascular resistance (PVR; B), pulse wave velocity (PWV; C), and via heart rate, peripheral vascular resistance and pulse wave velocity (D) in men.

	(mmHg/kg)	95% CI	*P*
**A. Mediator: heart rate**			
***Total effect***	***0.412***	***0.259–0.566***	***<0.001***
***Direct effect***	***0.361***	***0.209–0.513***	***<0.001***
***Indirect effect (HR)***	***0.051***	***0.014–0.098***	***—***
**B. Mediator: peripheral vascular resistance**			
***Total effect***	***0.412***	***0.258–0.565***	***<0.001***
***Direct effect***	***0.317***	***0.184–0.450***	***<0.001***
***Indirect effect (PVR)***	***0.095***	***0.029–0.169***	***—***
**C. Mediator: pulse wave velocity**			
***Total effect***	***0.405***	***0.250–0.560***	***<0.001***
***Direct effect***	***0.331***	***0.181–0.480***	***<0.001***
***Indirect effect (PWV)***	***0.074***	***0.021–0.144***	***—***
**D. Mediators: heart rate, peripheral vascular resistance and pulse wave velocity**			
***Total effect***	***0.397***	***0.241–0.554***	***<0.001***
***Direct effect***	***0.234***	***0.103–0.356***	***0.001***
***Total indirect effect***	***0.163***	***0.081–0.256***	***—***
***Indirect effect (HR)***	***0.048***	***0.012–0.092***	***—***
***Indirect effect (PVR)***	***0.087***	***0.024–0.158***	***—***
***Indirect effect (PWV)***	***0.029***	***0.001–0.072***	***—***

All models were adjusted for age and fat‐free mass. Significant effects are shown in bold italics. The indirect effects are considered significant when the corresponding confidence intervals do not include zero. CI, confidence interval.

**TABLE 7:** Mediation analysis of the association between fat mass and diastolic blood pressure via stroke volume (SV; A), heart rate (HR; B), peripheral vascular resistance (PVR; C), pulse wave velocity (PWV; D), and via heart rate, peripheral vascular resistance and pulse wave velocity (E) in women.

	Effect (mmHg/kg)	95% CI	*P*
**A. Mediator: stroke volume**			
***Total effect***	***0.532***	***0.351–0.713***	***<0.001***
***Direct effect***	***0.491***	***0.304–0.679***	***<0.001***
Indirect effect (SV)	0.040	−0.002–0.104	—
**B. Mediator: heart rate**			
***Total effect***	***0.526***	***0.345–0.707***	***<0.001***
***Direct effect***	***0.433***	***0.247–0.619***	***<0.001***
***Indirect effect (HR)***	***0.093***	***0.027–0.187***	—
**C. Mediator: peripheral vascular resistance**			
***Total effect***	***0.525***	***0.343–0.707***	***<0.001***
***Direct effect***	***0.342***	***0.176–0.507***	***<0.001***
***Indirect effect (PVR)***	***0.183***	***0.081–0.323***	—
**D. Mediator: pulse wave velocity**			
***Total effect***	***0.517***	***0.323–0.711***	***<0.001***
***Direct effect***	***0.342***	***0.181–0.502***	***<0.001***
***Indirect effect (PWV)***	***0.175***	***0.064–0.322***	—
**E. Mediators: heart rate, peripheral vascular resistance and pulse wave velocity**			
***Total effect***	***0.518***	***0.323–0.713***	***<0.001***
***Direct effect***	***0.195***	***0.036–0.353***	***0.016***
***Total indirect effect***	***0.323***	***0.177–0.493***	—
Indirect effect (HR)	0.044	−0.009–0.111	—
***Indirect effect (PVR)***	***0.150***	***0.060–0.272***	—
***Indirect effect (PWV)***	***0.130***	***0.040–0.242***	—

All models were adjusted for age and fat‐free mass. Significant effects are shown in bold italics. The indirect effects are considered significant when the corresponding confidence intervals do not include zero. CI, confidence interval.

Appendix Tables A1, A2, A3, A4, A5, A6 show the results of multivariable and mediation analyses after additional adjustment for height. In men, these analyses confirmed the main findings; however, in the multiple‐mediator model examining the FFM–SBP relationship, the total indirect effect of CO and PVR was significant and positive, indicating that the positive mediation of the FFM–SBP association via CO was only partially offset by the negative mediation via PVR (Appendix Table A3D). In women, the main results remained unchanged; however, in the multiple‐mediator models, the indirect effect via PWV was larger than that via PVR in the FM–SBP as well as in the FM–DBP associations in the height‐adjusted models (Appendix Tables A4E and A6E, respectively).

## 4 DISCUSSION

Our study examined the sex‐specific haemodynamic links between body composition parameters and BP in young adults. Beyond the well‐established positive association between BMI and both SBP and DBP observed in both sexes, we demonstrated that FFM is the dominant body composition correlate of SBP in young men, while FM plays a similar role in young women. In men, the positive association between FFM and SBP is driven in part by CO, while this effect is partially offset by opposing influences through PVR. In contrast, in women, the positive FM–SBP association is predominantly mediated by vascular pathways, including PVR and PWV. In both sexes, FM is the dominant body composition parameter associated with DBP. The positive association between FM and DBP is positively mediated by PVR and PWV in both sexes, and additionally by HR in men.

A limited number of earlier, smaller studies focused on the associations between body composition parameters and BP, as well as haemodynamic variables. Daniels et al. examined children and adolescents aged 6–17 years and showed that lean body mass was the dominant body composition correlate of SBP and DBP. In addition, both lean body mass and fat mass were positively associated with CO and SV, and negatively associated with PVR (Daniels et al., 1996). However, sex‐dependent differences in the haemodynamic mechanisms underlying the body composition–BP association were not specifically examined. Hunt et al. investigated 103 healthy sedentary adults (25 men and 78 women), and reported that FFM was positively associated with DBP, CO and SV, negatively associated with PVR, and there was no significant association between FFM and SBP (Hunt et al., 1998). They reported results of univariate analyses, and they did not show results about FM‐related associations. Based on our findings, the predominance of female participants in that study – among whom FM appears to be the dominant correlate of SBP – may have contributed to the absence of a detectable FFM–SBP association. In line with the results of both Daniels et al. and Hunt et al., we also observed significant positive associations between FFM and CO as well as SV, together with a negative association between FFM and PVR, in both men and women. As FFM is a major determinant of resting energy expenditure (Cunningham, 1991), and exhibits substantially higher metabolic demand than FM (Wang et al., 2010), these associations are physiologically plausible. Larger FFM requires increased peripheral perfusion to meet tissue oxygen and substrate demands, resulting in higher CO and SV, and lower PVR. The inverse association between FFM and PVR may in part reflect a larger vascular bed at higher FFM, with a greater number of resistance vessels arranged in parallel. Importantly, however, the relative importance of these FFM‐related mechanisms shows significant sex‐related differences based on our results. In men, FFM was the major determinant of SBP, and according to our age‐ and height‐adjusted parallel mediator models, increases in FFM were associated with higher CO, an effect only partially offset by a concomitant reduction in PVR. In addition, a significant direct positive FFM–SBP association remained that was not explained by the haemodynamic mechanisms assessed in our study, warranting further investigation. In contrast, FFM was not an independent correlate of SBP in women, suggesting that the mentioned FFM‐related physiological changes largely counterbalance each other. Instead, FM was the dominant independent correlate of SBP in women, with mediation analysis indicating that higher FM increases SBP via increased PVR and increased PWV. Together, these findings suggest that adipose tissue accumulation in women is associated predominantly with alterations in the resistance vasculature and increased aortic stiffness. These results are in line with our previous findings in The Enigma Study, which reported that hypertension was associated with a cardiac phenotype in men, and a vascular phenotype in women (Nardin et al., 2018).

More recently, our study based on the Anglo‐Cardiff Collaboration Trial (ACCT) cohort demonstrated that in young men, isolated systolic hypertension is the most prevalent form of hypertension, and in young women, isolated diastolic and systolic diastolic hypertension are the dominant forms of hypertension (Cseh et al., 2025). These results underline the importance of examining haemodynamic links between body composition and DBP, especially in young women. In both sexes, increased FM was associated with increased DBP in our study, and predominantly vascular alterations – increased PVR and PWV – mediated this association. The positive association of FM with PVR and PWV may reflect the vascular effects of adipose tissue. Adipocyte hypertrophy and adipose tissue dysfunction have been associated with sympathetic nervous system and renin–angiotensin–aldosterone system activation, inflammation, increased oxidative stress and endothelial dysfunction, which may favour increased vascular tone, and consequently increased vascular resistance and stiffness (Clayton et al., 2023; DeMarco et al., 2014). These findings are in line with the results of a recent meta‐analysis of 21,215 adults that showed a significant positive association between FM and PWV (Nikoohemmat et al., 2025), and with the results of the Dallas Heart Study (*n* = 2,710) that showed a significant positive association between visceral adipose tissue mass and systemic vascular resistance (Neeland et al., 2013).

Yu et al., examining a large cohort (*n* = 13,618) showed that FFM was the dominant contributor to SBP in children and adolescents (Yu et al., 2024). However, the difference in FM between boys and girls was smaller than in our adult population, in which men and women differ markedly, with women naturally having higher FM%, in line with previous findings (Coin et al., 2008). Based on our current and recent results, in young adults, the effects of FM‐related vascular alterations on SBP and DBP are very important in women; however, the significance of these FM‐related vascular alterations in men is less pronounced. The absence of an independent association between FM and SBP in men raises the question of whether male participants in our study may have been unusually lean and thus less representative of a healthy young population. Coin et al. examined 1866 healthy Italian adults using dual‐energy X‐ray absorptiometry (DXA) to establish age‐ and sex‐specific reference values for body composition parameters (Coin et al., 2008). In that study, FM% reference values were 16.9 ± 4.9% for men aged 20–29 years, and 20.8 ± 10.0% for men aged 30–39 years. In comparison, men aged 18–40 years in our population had a FM% of 18.5 ± 7.4%. Similarly, FM% reference values were 30.8 ± 10.2% for women aged 20–29 years, and 32.4 ± 16.4% for women aged 30–39 years, while women aged 18–40 years in our population had an FM% of 31.0 ± 9.0%. We acknowledge that although the body composition measures obtained using bioelectrical impedance analysis and DXA demonstrated strong correlation in previous studies, they are not directly interchangeable (Chen et al., 2016; Feng et al., 2024; Wahrlich et al., 2025). Since bioelectrical impedance analysis tended to underestimate FM or FM% relative to DXA in these studies, the broadly comparable FM% values provide reassurance that the male participants in our study were unlikely to have been unusually lean.

Weight gain typically reflects increases in FM, and also in FFM (Forbes & Welle, 1983), and weight loss can comprise highly variable reductions in FM and FFM depending on the nature of the intervention (Karakasis et al., 2025; Willoughby et al., 2018). Even the use of similar types of new, highly effective and widely available anti‐obesity drugs can result in differing degrees of FM and FFM reduction (Look et al., 2025; Wilding et al., 2021). A detailed understanding of the haemodynamic mechanisms underlying the body composition–BP association and their changes during weight gain and weight loss may facilitate more precise tailoring of antihypertensive therapy in individuals with increased adiposity according to the dominant underlying haemodynamic abnormalities.

Since the majority of the pharmacological weight loss trials have been conducted in populations older than our study cohort, large longitudinal interventional studies that concurrently monitor body composition, BP, and mediating haemodynamic mechanisms are particularly needed in young adults. Such studies may help improve the management of obesity‐related hypertension during emerging adulthood, a sensitive developmental period in which early interventions may yield substantial long‐term cardiovascular benefits (Scott et al., 2025).

We must acknowledge several limitations of our study. The cross‐sectional nature of the study limits inference regarding causality; therefore, longitudinal studies are needed to determine the temporal relationship between alterations in body composition, associated haemodynamic mechanisms, and subsequent changes in BP. In addition, our results did not provide information on fat mass distribution. Since previous results showed that peripheral and central fat mass accumulation might have different effects on BP (Malden et al., 2019; Yano et al., 2016; Zhao et al., 2022), it is also possible that the underlying haemodynamic mechanisms differ according to fat distribution. Furthermore, hydration status was not standardized before bioelectrical impedance analysis, which might have introduced some variability into our body composition estimates (Dixon et al., 2009). We also acknowledge that physical activity might influence body composition, BP and the haemodynamic mechanisms linking body composition to BP, and the absence of physical activity data in this analysis limited our ability to account for its potential confounding effects. Additionally, as the majority of our participants were of White ethnic origin, future studies should include ethnically more diverse populations to improve generalizability. Finally, our parallel mediator models included candidate mediators that are not entirely independent; thus, the results should be interpreted with caution. However, as the direction of effects in the multiple‐mediator models was consistent with those obtained from the multivariable linear regression and single‐mediator analyses, we believe that the overall conclusions of our study are robust.

### 4.1 Conclusion

Our study demonstrated substantial sex‐related differences in the haemodynamic mechanisms underlying the association between body composition and BP in young adults. While FFM is the dominant body composition parameter associated with SBP in men, FM plays the dominant role in women. In both sexes, FM is the dominant body composition correlate of DBP. In height‐adjusted mediation models, the positive FFM–SBP association in men is partly mediated by the positive effect via CO, which is partially offset by the negative effect via PVR. The positive FM–SBP association in women and the positive FM–DBP association in both sexes are driven predominantly by vascular mechanisms, as reflected by positive effects via PVR and PWV. A schematic summary of our main results is presented in Figure 2. These findings provide important insights into the physiology of adiposity‐related BP changes and may help inform the optimization of management strategies for obesity‐related hypertension.

**FIGURE 2:**
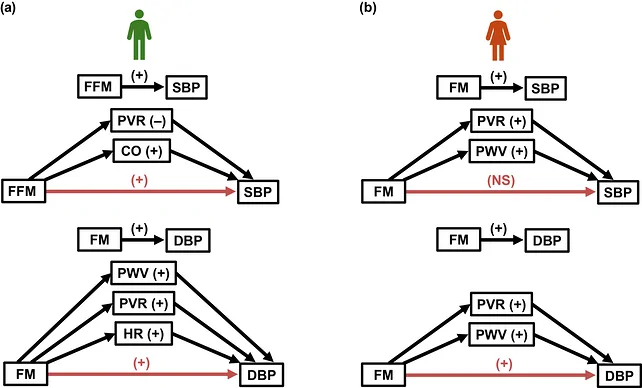
Schematic summary of regression and mediation models examining the associations between body composition parameters and blood pressure parameters in men (a) and women (b). CO, cardiac output; DBP, diastolic blood pressure; FFM, fat‐free mass; FM, fat mass; HR, heart rate; PVR, peripheral vascular resistance; PWV, pulse wave velocity; SBP, systolic blood pressure.

## AUTHOR CONTRIBUTIONS

Conceptualization: Carmel M. McEniery, Jessica E. Middlemiss and Ian B Wilkinson. Design and experimental protocol: Carmel M. McEniery and Ian B. Wilkinson. Data collection: Domonkos Cseh, Jessica E. Middlemiss, Shikai Yu, Gisele J. Bentley, Abbie Williams, Yasmin, Barry J. McDonnell, Christopher J. A. Pugh and Carmel M. McEniery. Data curation: Domonkos Cseh, Jessica E. Middlemiss and Carmel M. McEniery. Data analysis: Domonkos Cseh and Carmel M. McEniery. Writing – original draft preparation: Domonkos Cseh and Carmel M. McEniery. Writing – review and editing: Domonkos Cseh, Jessica E. Middlemiss, Shikai Yu, Gisele J. Bentley, Abbie Williams, Yasmin, Barry J. McDonnell, Christopher J. A. Pugh, Ian B. Wilkinson and Carmel M. McEniery. All authors approved the final version of the manuscript and agree to be accountable for all aspects of the work in ensuring that questions related to the accuracy or integrity of any part of the work are appropriately investigated and resolved. All persons designated as authors qualify for authorship, and all those who qualify for authorship are listed.

## CONFLICT OF INTEREST

J.E.M. was employed at University of Cambridge when the study was conducted, but now works at AstraZeneca, Cambridge, UK. The other authors have nothing to declare.

## GENERATIVE AI STATEMENT

All authors acknowledge that during the preparation of this manuscript, a generative AI toll (Chat GPT‐5.6) was used for language refinement and editing. No generative AI tools were used to generate scientific data.

## Data Availability

The data that support the findings of this study are available from the corresponding author upon reasonable request.

## 1

**TABLE A1:** Multivariable linear regression models examining the associations between body composition parameters and systolic blood pressure (A), diastolic blood pressure (B), cardiac output (C), stroke volume (D), heart rate (E), peripheral vascular resistance (F), and pulse wave velocity (G) in men.

	B	β	Unique *R* ^2^ (%)	*P*
**A. Systolic blood pressure**				
Adjusted *R* ^2 ^= 0.156				
***Age***	***0.323***	***0.160***	***2.0***	***0.019***
Fat mass	0.112	0.079	0.4	0.306
***Fat‐free mass***	***0.462***	***0.348***	***4.9***	***<0.001***
Height	−0.158	−0.099	0.5	0.253
**B. Diastolic blood pressure**				
Adjusted *R* ^2 ^= 0.353				
***Age***	***0.655***	***0.380***	***11.4***	***<0.001***
***Fat mass***	***0.387***	***0.318***	***6.2***	***<0.001***
Fat‐free mass	0.003	0.002	0.0	0.977
Height	−0.119	−0.087	0.4	0.248
**C. Cardiac output**				
Adjusted *R* ^2 ^= 0.274				
***Age***	−***0.090***	−***0.326***	***8.4***	***<0.001***
Fat mass	−0.009	−0.048	0.1	0.499
***Fat‐free mass***	***0.099***	***0.546***	***12.3***	***<0.001***
Height	−0.017	−0.078	0.3	0.328
**D. Stroke volume**				
Adjusted *R* ^2 ^= 0.265				
***Age***	−***0.986***	−***0.214***	***3.6***	***<0.001***
Fat mass	−0.404	−0.125	0.9	0.085
***Fat‐free mass***	***1.659***	***0.546***	***12.3***	***<0.001***
Height	−0.049	−0.013	0.0	0.868
**E. Heart rate**				
Adjusted *R* ^2 ^= 0.022				
Age	−0.119	−0.074	0.4	0.312
***Fat mass***	***0.262***	***0.232***	***3.3***	***0.006***
Fat‐free mass	−0.180	−0.171	1.2	0.098
Height	0.044	0.034	0.1	0.719
**F. Peripheral vascular resistance**				
Adjusted *R* ^2 ^= 0.315				
***Age***	***20.383***	***0.430***	***14.6***	***<0.001***
***Fat mass***	***4.835***	***0.145***	***1.3***	***0.039***
***Fat‐free mass***	−***12.037***	−***0.385***	***6.1***	***<0.001***
Height	−1.363	−0.036	0.1	0.646
**G. Pulse wave velocity**				
Adjusted *R* ^2 ^= 0.289				
***Age***	***0.070***	***0.413***	***13.8***	***<0.001***
***Fat mass***	***0.024***	***0.202***	***2.6***	***0.005***
Fat‐free mass	0.005	0.042	0.1	0.637
Height	0.003	0.023	0.0	0.775

Significant predictors are shown in bold italics.B, unstandardised regression coefficient; β, standardised regression coefficient.

**TABLE A2:** Multivariable linear regression models examining the associations between body composition parameters and systolic blood pressure (A), diastolic blood pressure (B), cardiac output (C), stroke volume (D), heart rate (E), peripheral vascular resistance (F), and pulse wave velocity (G) in women.

	B	β	Unique *R* ^2^ (%)	*P*
**A. Systolic blood pressure**				
Adjusted *R* ^2 ^= 0.165				
Age	0.203	0.079	0.6	0.252
***Fat mass***	***0.404***	***0.336***	***4.4***	***0.002***
Fat‐free mass	0.258	0.096	0.3	0.413
Height	−0.121	−0.059	0.2	0.479
**B. Diastolic blood pressure**				
Adjusted *R* ^2 ^= 0.240				
Age	0.260	0.122	1.4	0.063
***Fat mass***	***0.481***	***0.485***	***9.2***	***<0.001***
Fat‐free mass	−0.100	−0.045	0.1	0.688
Height	−0.165	−0.097	0.6	0.220
**C. Cardiac output**				
Adjusted *R* ^2 ^= 0.222				
Age	−0.023	−0.095	0.8	0.156
Fat mass	0.000	−0.011	0	0.995
***Fat‐free mass***	***0.085***	***0.337***	***3.6***	***0.003***
***Height***	***0.042***	***0.219***	***3.0***	***0.007***
**D. Stroke volume**				
Adjusted *R* ^2 ^= 0.208				
Age	0.113	0.030	0.1	0.655
***Fat mass***	−***0.435***	−***0.247***	***2.4***	***0.017***
***Fat‐free mass***	***1.683***	***0.425***	***5.8***	***<0.001***
***Height***	***0.672***	***0.224***	***3.2***	***0.006***
**E. Heart rate**				
Adjusted *R* ^2 ^= 0.088				
Age	−0.186	−0.100	0.9	0.164
***Fat mass***	***0.343***	***0.397***	***6.2***	***<0.001***
Fat‐free mass	−0.328	−0.170	0.9	0.168
Height	−0.161	−0.109	0.8	0.210
**F. Peripheral vascular resistance**				
Adjusted *R* ^2 ^= 0.190				
Age	6.887	0.122	1.4	0.076
***Fat mass***	***6.664***	***0.250***	***2.5***	***0.018***
***Fat‐free mass***	−***15.465***	−***0.259***	***2.2***	***0.026***
***Height***	−***12.435***	−***0.277***	***4.9***	***0.001***
**G. Pulse wave velocity**				
Adjusted *R* ^2 ^= 0.149				
***Age***	***0.032***	***0.212***	***4.2***	***0.003***
***Fat mass***	***0.022***	***0.291***	***3.4***	***0.007***
Fat‐free mass	0.005	0.029	0.0	0.811
Height	−0.001	−0.005	0.0	0.955

Significant predictors are shown in bold italics.B, unstandardised regression coefficient; β, standardised regression coefficient.

**TABLE A3:** Mediation analyses of the association between fat‐free mass and systolic blood pressure via cardiac output (CO; A), stroke volume (SV; B), peripheral vascular resistance (PVR; C), and via cardiac output and peripheral vascular resistance (D) in men.

	(mmHg/kg)	95% CI	*P*
**A. Mediator: cardiac output**			
***Total effect***	***0.462***	***0.215–0.709***	***<0.001***
***Direct effect***	***0.334***	***0.070–0.599***	***0.014***
***Indirect effect (CO)***	***0.128***	***0.019–0.253***	***—***
**B. Mediator: stroke volume**			
***Total effect***	***0.462***	***0.215–0.709***	***<0.001***
***Direct effect***	***0.477***	***0.209–0.744***	***<0.001***
Indirect effect (SV)	−0.015	−0.126–0.079	***—***
**C. Mediator: peripheral vascular resistance**			
***Total effect***	***0.467***	***0.219–0.714***	***<0.001***
***Direct effect***	***0.612***	***0.362–0.862***	***<0.001***
***Indirect effect (PVR)***	−***0.145***	−***0.263–***−***0.049***	***—***
**D. Mediators: cardiac output and peripheral vascular resistance**			
***Total effect***	***0.467***	***0.219–0.714***	***<0.001***
***Direct effect***	***0.277***	***0.072–0.483***	***0.009***
***Total indirect effect***	***0.200***	***0.006–0.375***	***—***
***Indirect effect (CO)***	***0.779***	***0.518–1.068***	***—***
***Indirect effect (PVR)***	−***0.590***	−***0.841–***−***0.360***	***—***

All models were adjusted for age, fat mass and height. Significant effects are shown in bold italics. The indirect effects are considered significant when the corresponding confidence intervals do not include zero. CI, confidence interval.

**TABLE A4:** Mediation analyses of the association between fat mass and systolic blood pressure via stroke volume (SV; A), heart rate (HR; B), peripheral vascular resistance (PVR; C), pulse wave velocity (PWV; D), and via heart rate, peripheral vascular resistance and pulse wave velocity (E) in women.

	Effect (mmHg/kg)	95% CI	*P*
**A. Mediator: stroke volume**			
***Total effect***	***0.405***	***0.152–0.657***	***0.002***
***Direct effect***	***0.404***	***0.147–0.661***	***0.002***
Indirect effect (SV)	0.000	−0.040–0.057	***—***
**B. Mediator: heart rate**			
***Total effect***	***0.399***	***0.148–0.649***	***0.002***
***Direct effect***	***0.336***	***0.078–0.593***	***0.011***
***Indirect effect (HR)***	***0.063***	***0.0002–0.159***	***—***
**C. Mediator: peripheral vascular resistance**			
***Total effect***	***0.383***	***0.130–0.636***	***0.003***
***Direct effect***	***0.252***	***0.019–0.485***	***0.034***
***Indirect effect (PVR)***	***0.131***	***0.022–0.292***	***—***
**D. Mediator: pulse wave velocity**				
***Total effect***	***0.306***	***0.040–0.572***	***0.024***	
Direct effect	0.103	−0.123–0.330	0.369	
***Indirect effect (PWV)***	***0.203***	***0.062–0.382***	***—***	
**E. Mediators: heart rate, peripheral vascular resistance and pulse wave velocity**				
***Total effect***	***0.307***	***0.037–0.577***	***0.026***	
Direct effect	0.013	−0.213–0.239	0.911	
***Total indirect effect***	***0.294***	***0.120–0.514***	***—***	
Indirect effect (HR)	0.017	−0.049–0.092	***—***	
***Indirect effect (PVR)***	***0.109***	***0.024–0.244***	***—***	
***Indirect effect (PWV)***	***0.169***	***0.050–0.323***	***—***	

All models were adjusted for age, fat‐free mass and height. Significant effects are shown in bold italics. The indirect effects are considered significant when the corresponding confidence intervals do not include zero. CI, confidence interval.

**TABLE A5:** Mediation analyses of the association between fat mass and diastolic blood pressure via heart rate (HR; A), peripheral vascular resistance (PVR; B), pulse wave velocity (PWV; C), and via heart rate, peripheral vascular resistance and pulse wave velocity (D) in men.

	Effect (mmHg/kg)	95% CI	*P*
**A. Mediator: heart rate**			
***Total effect***	***0.391***	***0.229–0.553***	***<0.001***
***Direct effect***	***0.337***	***0.177–0.498***	***<0.001***
***Indirect effect (HR)***	***0.054***	***0.013–0.105***	***—***
**B. Mediator: peripheral vascular resistance**			
***Total effect***	***0.379***	***0.218–0.540***	***<0.001***
***Direct effect***	***0.291***	***0.152–0.430***	***<0.001***
***Indirect effect (PVR)***	***0.088***	***0.017–0.166***	***—***
**C. Mediator: pulse wave velocity**			
***Total effect***	***0.376***	***0.213–0.539***	***<0.001***
***Direct effect***	***0.299***	***0.142–0.456***	***<0.001***
***Indirect effect (PWV)***	***0.077***	***0.022–0.148***	***—***
**D. Mediators: heart rate, peripheral vascular resistance and pulse wave velocity**			
***Total effect***	***0.380***	***0.215–0.544***	***<0.001***
***Direct effect***	***0.220***	***0.082–0.357***	***0.002***
***Total indirect effect***	***0.160***	***0.073–0.259***	***—***
***Indirect effect (HR)***	***0.049***	***0.011–0.098***	***—***
***Indirect effect (PVR)***	***0.082***	***0.014–0.157***	***—***
***Indirect effect (PWV)***	***0.030***	***0.001–0.076***	***—***

All models were adjusted for age, fat‐free mass and height. Significant effects are shown in bold italics. The indirect effects are considered significant when the corresponding confidence intervals do not include zero. CI, confidence interval.

**TABLE A6:** Mediation analyses of the association between fat mass and diastolic blood pressure via stroke volume (SV; A), heart rate (HR; B), peripheral vascular resistance (PVR; C), pulse wave velocity (PWV; D), and via heart rate, peripheral vascular resistance and pulse wave velocity (E) in women.

	Effect (mmHg/kg)	95% CI	*P*
**A. Mediator: stroke volume**			
***Total effect***	***0.488***	***0.290–0.686***	***<0.001***
***Direct effect***	***0.463***	***0.262–0.664***	***<0.001***
Indirect effect (SV)	0.025	−0.007–0.078	***—***
**B. Mediator: heart rate**			
***Total effect***	***0.477***	***0.280–0.674***	***<0.001***
***Direct effect***	***0.398***	***0.198–0.597***	***<0.001***
***Indirect effect (HR)***	***0.079***	***0.018–0.168***	***—***
**C. Mediator: peripheral vascular resistance**			
***Total effect***	***0.478***	***0.278–0.677***	***<0.001***
***Direct effect***	***0.359***	***0.182–0.537***	***<0.001***
***Indirect effect (PVR)***	***0.118***	***0.022–0.256***	***—***
**D. Mediator: pulse wave velocity**				
***Total effect***	***0.459***	***0.247–0.671***	***<0.001***	
***Direct effect***	***0.286***	***0.112–0.460***	***0.001***	
***Indirect effect (PWV)***	***0.174***	***0.050–0.323***	***—***	
**E. Mediators: heart rate, peripheral vascular resistance and pulse wave velocity**				
***Total effect***	***0.468***	***0.253–0.682***	***<0.001***	
***Direct effect***	***0.192***	***0.024–0.360***	***0.026***	
***Total indirect effect***	***0.276***	***0.122–0.464***	***—***	
Indirect effect (HR)	0.041	−0.011–0.106	***—***	
***Indirect effect (PVR)***	***0.101***	***0.023–0.214***	***—***	
***Indirect effect (PWV)***	***0.135***	***0.040–0.258***	***—***	

All models were adjusted for age, fat‐free mass and height. Significant effects are shown in bold italics. The indirect effects are considered significant when the corresponding confidence intervals do not include zero. CI, confidence interval.
